# Circulating PCSK9 levels and 2-hPG are positively correlated in metabolic diseases in a Chinese Han population

**DOI:** 10.1186/s12944-018-0658-z

**Published:** 2018-01-17

**Authors:** Wen Guo, Yingyun Gong, Yong Gu, Zhenzhen Fu, Hongqi Fan, Beibei Gao, Xiaohui Zhu, Jinxiang Fu, Yang Zhao, Min Sun, Xing Liu, Xian-Cheng Jiang, Tao Yang, Hongwen Zhou

**Affiliations:** 1 Department of Endocrinology and Metabolism, The First Affiliated Hospital of Nanjing Medical University, 300 Guangzhou Road, Nanjing, 210029 China; 2 Department of Endocrinology, Nanjing Municipal Hospital for Governmental Organizations, Nanjing, 210018 China; 3 School of Public Health, Nanjing Medical University, Nanjing, 210029 China; 4 Department of Endocrinology, Ansteel Group Hospital, Anshan, 114001 China; 5 Department of Cell Biology, SUNY Downstate Medical Center, New York, 11203 USA

**Keywords:** Proprotein convertase subtilisin/kexin type 9, 2-h postchallenge plasma glucose, Lipid

## Abstract

**Background:**

Proprotein convertase subtilisin/kexin type 9 (PCSK9), which plays a crucial role in lipoprotein metabolism, has been also regarded as an important marker for atherosclerosis. Available evidence indicated that 2-h postchallenge plasma glucose (2-hPG) could be another biomarker for atherosclerosis. However, currently the association between circulating PCSK9 and 2-hPG remains unclear. Here, we explored this potential link in a Chinese Han population.

**Methods:**

Totally, 600 Chinese Han subjects from Nanjing district, China, were enrolled for the 75-g oral glucose tolerance test (OGTT), and they included normal glucose tolerance (NGT, *n* = 200), impaired glucose regulation (IGR, n = 200), and type 2 diabetes (T2DM, n = 200). Anthropometric and biochemical determinations such as serum lipid measurements were made. A sandwich ELISA assay was performed to measure serum PCSK9 levels in all subjects.

**Results:**

Serum PCSK9 concentrations were higher in IGR group (77.63 ± 28.14 ng/ml) and T2DM group (90.62 ± 39.96 ng/ml) than in NGT group (65.33 ± 32.68 ng/ml), and it was significantly higher in T2DM group than in IGR group (*p* < 0.01). Serum PCSK9 levels positively correlated with 2-hPG and LDL-C in all subgroups, but presented a positive correlation with fasting blood glucose (FBG) only in T2DM group. Using multiple regression model analysis, we also found that PCSK9 levels closely correlated with 2-hPG in all tested groups. According to multinomial logistic regression analysis, PCSK9 levels positively correlated with T2DM (OR = 1.017[1.010–1.025], *p* < 0.001) even after adjustment for lipid levels. Moreover, in subjects with normal FBG level, 2-hPG gradually and significantly increased across PCSK9 tertiles (6.68 ± 2.01, 7.48 ± 2.10 and 8.27 ± 2.41 mmol/L, respectively, *p* < 0.01); however, in subjects with normal 2-hPG levels, no such difference was observed.

**Conclusions:**

PCSK9 levels increase as glucose metabolism deteriorated. Serum PCSK9 levels positively correlated with 2-hPG in patients with metabolic diseases.

## Background

Type 2 diabetes (T2DM) is generally accompanied by high triglyceride (TG) levels, low high- density lipoprotein cholesterol (HDL-C) levels and increased small and dense low-density lipoprotein (LDL) levels, which all confer high risk of cardiovascular disease (CVD) [1]. Small and dense LDL particle numbers are of particular concern among these risk factors. Serum LDL particles are mainly removed by the hepatic LDL receptor (LDLR). Proprotein convertase subtilisin/kexin type 9 (PCSK9), a member of the pre-proprotein convertase family of zymogens, is synthesized primarily in the liver. It has been proven that PCSK9 increases the degradation of LDLR on the surface of hepatocytes, thus decreases the uptake of LDL-C via LDLR, leading to the increase of plasma LDL-C levels [2]. Gain-of-function mutations in PCSK9 can cause hypercholesterolemia leading to premature coronary heart disease, whereas loss-of-function mutations are associated with reduced plasma levels of LDL-C and the risk of coronary heart disease [3]. Since elevated LDL-C has long been established as a predominant risk factor of CVD, manipulating PCSK9 concentration has been considered as the new promising strategy for the treatment of CVD. Meanwhile, several studies recently indicated that the harmful effects of PCSK9 on vascular biology might be mediated by mechanism occurring via LDLR-independent pathways [4]. For example, PCSK9 directly correlates with other well-known CVD risk factors, including TG levels, glucose metabolism, and insulin resistance [5].

Evidence so far suggests that high blood glucose level may be a risk factor for CVD in T2DM patients. The DECODE study demonstrated that 2-h postchallenge plasma glucose (2-hPG) is a better risk predictor of cardiovascular disease than fasting blood glucose (FBG) [6]. The level of 2-hPG is an independent risk factor of carotid intima-media thickness progression in T2DM [7] as well as in subjects with normal glucose tolerance [8].

Besides lipid metabolism and atherosclerosis, several studies have emphasized a possible link between PCSK9 and glucose metabolism. The Dallas Heart Study [9] and our previous study [10] also showed that PCSK9 levels positively correlated with fasting glucose. So far, there are no data concerning the association between circulating PCSK9 concentration and 2-hPG. Considering the importance of 2-hPG in CVD and the crucial role of PCSK9 in the development of CVD, we therefore aimed to investigate the association of PCSK9 and 2-hPG in metabolic diseases in a Chinese Han population.

## Methods

### Subjects

A population-based cross-sectional study in Gulou district, Nanjing, China was conducted from June to December in 2011. Based on the 75-g oral glucose tolerance test (OGTT), 2986 subjects were assigned into three different groups, namely normal glucose tolerance (NGT, *N* = 1000), impaired glucose regulation (IGR, *N* = 1012), including impaired fasting glucose (IFG) and/or impaired glucose tolerance (IGT), and T2DM (*N* = 974). For further analysis, 200 age- and gender- matched participants were selected from each group to measure serum PCSK9 concentration, thus 600 subjects aged 40 years or older were finally enrolled, consisting of 248 males and 352 females. All subjects signed informed consent forms. Exclusion criteria included diabetic ketosis, hyperthyroidism, history of usage of statin lipid-lowering drugs, and liver, kidney or other diseases associated with lipid metabolism disorders. The project was approved by the Ethical Committee of the First Affiliated Hospital of Nanjing Medical University (2011-SR-128).

### Blood sample collection and storage

All subjects underwent an OGTT in the morning after overnight fasting, blood samples were collected during the course of OGTT, i.e. 0 and 120 min after glucose challenge. Blood samples were centrifuged at 4 °C (3,000 rpm, 15 min), and serum were separated and stored at −80 °C.

### Physical examination and biochemical tests

Body weight, height, waist circumference (WC) and blood pressure were measured in accordance with international standards. Plasma glucose, total cholesterol (TC), TG, LDL-C and HDL-C concentrations were measured by enzymatic methods (Chemistry Analyzer Au2700, Olympus Medical Engineering Company, Japan). Serum insulin concentration was assessed in duplicate by a highly specific radioimmunoassay, Phadebas insulin test (Pharmacia, Uppsala, Sweden), as previously reported [11]. Insulin resistance homeostasis model assessment index (HOMA-IR) was calculated as fasting insulin [IU/mL] * fasting blood glucose [mmol/L] / 22.5).

### Serum PCSK9 concentration measured by ELISA

Fasting blood samples were collected to measure serum PCSK9 levels in all 600 subjects. Serum PCSK9 concentration was measured by a sandwich ELISA assay as previously described [10, 12]. Briefly, 96-well plates were pre-coated with anti-PCSK9 antibody overnight, then incubated with standard recombinant PCSK9 protein or human serum samples, HRP labeled anti-PCSK9 antibody, development substrate and stop solution were accordingly incubated. Lastly, the absorbance was detected at 450 nm. The limit of quantification was 1 ng/ml. The intra-assay and inter-assay coefficient of variation (CV) was 7.9% and 10.2%, respectively.

### Statistical analysis

Data are expressed as mean ± SD. Differences among groups were tested by one-way ANOVA with Bonferroni correction for pair wise comparisons. The relationship between PCSK9 and other variables were analyzed by Spearman rank correlation. Specific multivariable stepwise regression models were built to determine the factors that were independent of the PCSK9 levels. We stratified the participants into three groups by the tertiles of 2-hPG in 284 subjects with normal FBG to investigate the association with 2-hPG and PCSK9. In the same way, we stratified the participants into three groups by the tertiles of FBG in 276 subjects with normal 2-hPG to investigate the association between FBG and PCSK9. Multinomial logistic regression was used to examine the association between glucose status (0 = NGT, 1 = IGR, and 2 = T2DM) as dependent variables with PCSK9 adjusted for the covariate variables including age, sex, blood pressure, BMI, WC and lipid levels. Odds ratios and 95% confidence intervals (95% CI) were determined. Data were analyzed by SPSS18.0 statistical software, with significance defined as *p* < 0.05 (two-sided).

## Results

### Participants characteristics and serum PCSK9 levels

Clinical and biochemical characteristics are listed in Table 1. Individuals with IGR or T2DM were characterized by higher blood pressure (*p* < 0.01), BMI (*p* < 0.01), heart rate (*p* < 0.01), TG (*p* < 0.01), FBG (*p* < 0.01), 2-hPG (*p* < 0.01) and 2 h–INS (*p* < 0.01), increased waist circumference (*p* < 0.01), hip circumference (*p* < 0.05), Waist-to-Hip ratio (*p* < 0.01), HOMA-IR (*p* < 0.01) than the NGT group. Those in T2DM group were characterized by higher LDL-C, fasting insulin (FINS) and lower HDL-C levels than in NGT, but there was no difference between participants with IGR and NGT. Subjects with IGR were characterized by higher TC than with NGT, and those with T2DM had higher TC than with NGT but without significant difference (*p* > 0.05). Serum PCSK9 levels were significantly higher in subjects with abnormal glucose tolerance than in those with normal glucose tolerance (*p* < 0.01), and were also significantly higher in T2DM subjects than in IGR subjects (*p* < 0.01). IGR subjects were classified into three groups based on WHO diagnostic criteria: 64 subjects with combined IFG/IGT, 72 subjects with IGT, and 74 subjects with IFG. Subjects with combined IFG/IGT or IGT only had higher PCSK9 levels than IFG subjects (89.73 ± 22.01, 83.97 ± 29.68, 61.86 ± 24.29 ng/ml, respectively, *p* < 0.01) but there was no significant difference between subjects with combined IFG/IGT and those with IGT only (Data not shown).

**Table 1:** The clinical and biochemical properties of 600 participants

	All (*n* = 600)	NGT (n = 200)	IGR (n = 200)	T2DM (n = 200)
Age (years)	59.49 ± 8.06	59.01 ± 7.43	59.63 ± 8.05	59.84 ± 8.67
Male/female	248/352	79/121	86/114	83/117
SBP (mmHg)	136.63 ± 20.38	129.79 ± 19.31	137.05 ± 18.06^b^	143.12 ± 21.51^b,d^
DBP (mmHg)	80.77 ± 12.04	77.33 ± 10.53	81.34 ± 11.39 ^b^	83.68 ± 13.24^b^
BMI (kg/m^2^)	24.75 ± 3.71	23.60 ± 3.41	25.46 ± 3.22^b^	25.19 ± 4.16^b^
WC (cm)	86.33 ± 9.32	83.67 ± 9.07	87.82 ± 9.30^b^	87.47 ± 9.05^b^
HC (cm)	97.13 ± 6.46	95.88 ± 6.32	97.90 ± 6.64 ^b^	97.60 ± 6.24 ^a^
WHR	0.89 ± 0.07	0.87 ± 0.07	0.89 ± 0.06 ^b^	0.89 ± 0.06^b^
HR (per min)	78.73 ± 11.95	74.79 ± 10.59	80.16 ± 11.52 ^b^	81.27 ± 12.69 ^b^
HDL-C (mmol/L)	1.30 ± 0.32	1.34 ± 0.33	1.33 ± 0.32	1.23 ± 0.31^b,d^
LDL-C (mmol/L)	2.91 ± 0.71	2.78 ± 0.66	2.94 ± 0.64	2.99 ± 0.81^a^
TC (mmol/L)	5.00 ± 0.95	4.86 ± 0.88	5.09 ± 0.84^a^	5.05 ± 1.09
TG (mmol/L)	1.76 ± 1.29	1.38 ± 0.71	1.77 ± 0.95^b^	2.12 ± 1.82^b,c^
FBG (mmol/L)	6.30 ± 1.55	5.37 ± 0.36	6.13 ± 0.54^b^	7.40 ± 2.18^b,d^
2-hPG (mmol/L)	9.61 ± 4.26	6.21 ± 0.91	8.22 ± 1.51^b^	14.40 ± 3.86^b,d^
FINS (μIU/ml)	12.71 ± 13.72	10.13 ± 6.54	12.97 ± 11.70	15.03 ± 19.37^b^
2 h–INS (μIU/ml)	74.94 ± 62.64	47.41 ± 37.54	76.94 ± 59.63^b^	100.46 ± 73.66^b,d^
HOMA-IR	3.9 ± 0.1	2.43 ± 1.58	3.52 ± 3.15 ^a^	4.97 ± 6.64 ^b,d^
PCSK9 (ng/ml)	77.86 ± 35.43	65.33 ± 32.68	77.63 ± 28.14^b^	90.62 ± 39.96^b,d^

*SBP* systolic blood pressure, *DBP* diastolic blood pressure, *BMI* body mass index, *WC* waist circumference, *HC* hip circumference, *WHR* waist-to-hip ratio, *HR* heart rate, *HDL-C* high-density lipoprotein cholesterol, *LDL-C* low-density lipoprotein cholesterol, *TC* total cholesterol, *TG* triglyceride, *FBG* fasting blood glucose, *2-hPG* 2-h postchallenge plasma glucose, *FINS* fasting insulin, *2 h–INS* 2-h postchallenge plasma insulin, *HOMA-IR* homeostasis model assessment-estimated insulin resistance index, *PCSK9* Proprotein convertase subtilisin/kexin type 9

Compared with NGT, ^a^*p* < 0.05, ^b^*p* < 0.01; Compared with IGR, ^c^*p* < 0.05, ^d^*p* < 0.01

### The relationship between PCSK9, blood glucose and other metabolic parameters

To examine the relationship between serum PCSK9 levels and other parameters, particularly 2-hPG, correlation analyses were performed (Table 2). Serum PCSK9 levels positively correlated with 2-hPG in all groups. The PCSK9 levels were also positively correlated with HC, HR, LDL-C and 2 h–INS in NGT group, and positively correlated with SBP, BMI, LDL-C, TC, FINS, 2 h–INS and HOMA-IR in IGR group. In T2DM group, PCSK9 levels positively correlated with LDL-C, TG, FBG, FINS and HOMA-IR. The correlation between 2-hPG and serum PCSK9 concentration remained significant (Standardized β = 0.332, *p* < 0.001) when SBP, BMI, WC, HC, HR, WHR, as well as FBG, LDL-C, TC, TG, FINS and HOMA-IR were further adjusted by multivariable stepwise regression. However, when 2-hPG was further adjusted, the correlation between FBG and PCSK9 was no longer statistically significant.

**Table 2:** Spearman rank correlation between serum PCSK9 and different covariates in groups with different glucose tolerance

	All (n = 600)		NGT (n = 200)		IGR (n = 200)		T2DM (n = 200)	
	r	*p*	r	*p*	r	*p*	r	*p*
Age(years)	0.086	0.035	0.105	0.138	0.113	0.112	0.034	0.629
SBP(mmHg)	0.113	0.006	−0.074	0.297	0.202	0.004	−0.012	0.866
DBP(mmHg)	0.016	0.695	−0.087	0.219	−0.007	0.922	−0.077	0.278
BMI(kg/m^2^)	0.180	<0.001	0.109	0.126	0.146	0.039	0.101	0.157
WC(cm)	0.131	0.001	0.068	0.343	0.051	0.476	0.094	0.190
HC(cm)	0.101	0.014	0.153	0.032	0.048	0.503	0.025	0.729
WHR	0.103	0.013	−0.028	0.701	0.038	0.590	0.120	0.095
HR	0.174	<0.001	0.171	0.016	0.101	0.153	0.039	0.582
HDL-C(mmol/L)	−0.042	0.309	−0.025	0.729	0.071	0.314	−0.038	0.590
LDL-C(mmol/L)	0.192	<0.001	0.194	0.006	0.201	0.004	0.145	0.040
TC(mmol/L)	0.134	0.001	0.100	0.159	0.164	0.021	0.136	0.055
TG(mmol/L)	0.165	0.001	0.047	0.505	0.055	0.443	0.166	0.019
FBG(mmol/L)	0.266	<0.001	−0.008	0.915	−0.039	0.583	0.223	0.001
2-hPG(mmol/L)	0.426	<0.001	0.215	0.002	0.512	0.001	0.323	<0.001
FINS(μIU/ml)	0.249	<0.001	0.096	0.175	0.214	0.002	0.243	0.001
2 h–INS(μIU/ml)	0.231	<0.001	0.149	0.035	0.306	<0.001	−0.007	0.916
HOMA-IR	0.294	<0.001	0.099	0.163	0.176	0.013	0.312	<0.001

*SBP* systolic blood pressure, *DBP* diastolic blood pressure, *BMI* body mass index, *WC* waist circumference, *HC* hip circumference, *WHR* waist-to-hip ratio, *HR* heart rate, *HDL-C* high-density lipoprotein cholesterol, *LDL-C* low-density lipoprotein cholesterol, *TC* total cholesterol, *TG* triglyceride, *FBG* fasting bloodglucose, *2-hPG* 2-h postchallenge plasma glucose, *FINS* fasting insulin, *2 h–INS* 2-hpostchallenge plasma insulin, *PCSK9* Preproteinconvertasesubtilisin/kexin type 9, *HOMA-IR* homeostasis model assessment-estimated insulin resistance index

^a^*p* < 0.05, ^b^*p* < 0.01

### 2-hPG across the tertiles of PCSK9 levels in subjects with normal FBG

**Fig. 1:**
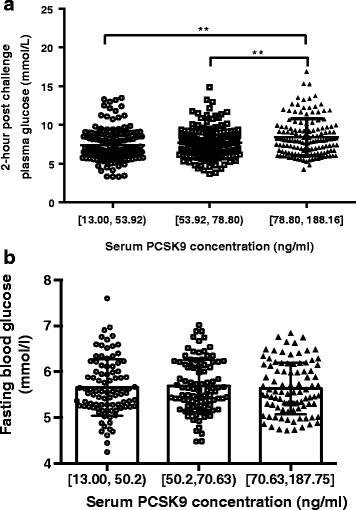
**a**: 2-hPG levels across the tertiles of serum PCSK9 concentration in 284 subjects with normal fasting glucose. **b**: Fasting blood glucose levels across the tertiles of serum PCSK9 concentration in 276 subjects with normal 2-hPG. 2-hPG, 2-h postchallenge plasma glucose; PCSK9, Proprotein convertase subtilisin/kexin type 9

To further validate the relationship between PCSK9 and 2-hPG independent of FBG, we stratified 284 participants with normal FBG (<6.0 mmol/L) into three groups based on the tertiles of serum PCSK9 concentration. We found that the upper PCSK9 level tertiles group had higher 2-hPG than the middle and lower PCSK9 level tertiles group (8.27 ± 2.41, 7.48 ± 2.10 and 6.68 ± 2.01 mmol/L, respectively, *p* < 0.01) (Fig. 1A). However, when we stratified 276 participants with normal 2-hPG (<7.8 mmol/L) into three groups by the tertiles of serum PCSK9 level, there were no differences in FBG levels among the three groups (5.65 ± 0.61, 5.69 ± 0.56 and 5.64 ± 0.56 mmol/L, respectively, *p* = 0.841) (Fig. 1B).

According to multinomial logistic regression analysis, when age, sex, BMI, HC, WC, WHR and heart rate were forced in the model, PCSK9 levels positively correlated with IGR (OR = 1.008[1.001–1.015], *p* = 0.019). However, after additional adjustment for lipid parameters, the relationship between PCSK9 and IGR was weak (OR = 1.007[1.000–1.014], *p* = 0.059). Serum PCSK9 levels positively correlated with T2DM (OR = 1.018[1.011–1.025], *p* < 0.001) when age, sex, BMI, HC, WC, WHR and heart rate were forced in the model and this relationship even existed when lipid levels were forced in the model (OR = 1.017[1.010–1.025], *p* < 0.001) (Table 3).

**Table 3:** Multinomial logistic analysis of the risk factors for T2DM

Variables	Model	IGR		T2DM	
		OR(95%CI)	P	OR(95%CI)	P
Age	1	0.995(0.775–1.277)	0.967	0.938 (0.723–1.216)	0.628
(10 years)	2	1.008 (0.782–1.300)	0.949	0.967 (0.743–1.259)	0.803
Sex	1	0.892 (0.577–1.379)	0.606	0.977 (0.620–1.541)	0.921
	2	0.874 (0.561–1.361)	0.551	1.019 (0.641–1.619)	0.936
BP	1	1.705 (1.084–2.680)	0.021	2.595 (1.627–4.139)	<0.001
(mmHg)	2	1.641 (1.037–2.596)	0.034	2.483 (1.549–3.982)	<0.001
BMI	1	2.411 (1.490–3.902)	<0.001	2.154 (1.309–3.545)	0.003
(kg/m^2^)	2	2.450 (1.505–3.986)	<0.001	2.044 (1.233–3.389)	0.006
WC	1	0.836 (0.424–1.648)	0.604	0.796 (0.395–1.604)	0.523
(cm)	2	0.906 (0.455–1.801)	0.778	0.809 (0.397–1.647)	0.558
HC	1	0.990 (0.942–1.040)	0.683	0.983 (0.933–1.036)	0.525
(cm)	2	0.984 (0.936–1.035)	0.526	0.978 (0.927–1.032)	0.416
WHR	1	1.026 (0.588–1.793)	0.927	1.279 (0.716–2.287)	0.406
	2	1.003 (0.572–1.760)	0.992	1.222 (0.679–2.200)	0.504
HR	1	1.040 (1.020–1.060)	<0.001	1.041 (1.021–1.062)	<0.001
(per min)	2	1.036 (1.016–1.057)	<0.001	1.039 (1.018–1.060)	<0.001
HDL-C	1	___	___	___	___
(mmol/l)	2	1.661 (1.023–2.698)	0.040	0.777 (0.462–1.304)	0.339
LDL-C	1	___	___	___	___
(mmol/L)	2	1.268 (0.661–2.436)	0.475	1.796 (0.894–3.608)	0.100
TC	1	___	___	___	___
(mmol/L)	2	1.012 (0.460–2.229)	0.976	0.942 (0.413–2.151)	0.887
TG	1	___	___	___	___
(mmol/L)	2	2.411 (1.266–4.591)	0.007	2.383 (1.242–4.572)	0.009
PCSK9	1	1.008 (1.001–1.015)	0.019	1.018 (1.011–1.025)	<0.001
(ng/ml)	2	1.007 (1.000–1.014)	0.059	1.017 (1.010–1.025)	<0.001

Model1: age, sex, BP, BMI, WC, HC, WHR, HR and PCSK9 were in the model

Model2: Model 1 plus HDL-C, LDL-C, TC, TG

*BP* blood pressure, *BMI* body mass index, *WC* waist circumference, *WHR* waist-to-hip ratio, *HR* heart rate, *HDL-C* high-density lipoprotein cholesterol, *LDL-C* low-density lipoprotein cholesterol, *TC* total cholesterol, *TG* triglyceride, *PCSK9* Preprotein convertase subtilisin/kexin type 9

## Discussion

The major finding of our study is that 2-hPG is positively correlated with PCSK9 independent of other metabolic parameters such as lipid parameters. The mechanism of this association is still unknown. Increasing evidence has suggested that 2-hPG is a stronger risk predictor than FBG for incident of coronary heart disease and CVD mortality [8, 13, 14]. The pathophysiology basis for the association between 2-hPG and coronary artery disease (CAD) is not fully understood. Others reported that there is a correlation between 2-hPG and some markers for low grade inflammation, such as plasminogen activator inhibitor and sensitive C-reactive protein (CRP) [15]. Another study has showed that there is a correlation between 2-hPG and pro-inflammatory markers such as tumor necrosis factor alpha [16]. Interestingly, there is also a linkage between PCSK9 and inflammation. LPS-induced inflammation resulted in a marked increase in hepatic PCSK9 mRNA levels, and knockdown of PCSK9 by small-interfering RNA (siRNA) suppressed the expression of pro-inflammatory genes [17, 18]. Serum PCSK9 levels positively correlated with white blood cell count (WBCC), a traditional and easy-to-measure marker of chronic low grade inflammation, in patients with stable coronary artery disease [19]. Plasma PCSK9 levels were also positively associated with platelet (PLT) count, another useful indicator associated with CVD, in patients with stable coronary artery disease [5]. Another study showed that there were positive associations of PCSK9 with inflammatory markers, such as fibrinogen and CRP in stable CAD status [20]. In the current study, we found that serum PCSK9 levels positively associated WBCC (*r* = 0.076, *p* < 0.01) and PLT count (*r* = 0.077, *p* < 0.01). Thus, the linkage between PCSK9 and 2-hPG could be inflammation. Whether PCSK9 could influence 2-hPG or vice versa deserves further study.

Another major finding of our study is that serum PCSK9 level increased as glucose metabolism deteriorated and it could be an independent predictor of T2DM (1.017[1.010–1.025]) even after adjustment for lipid levels. In our study, PCSK9 levels were higher in T2DM group compared to IGR and NGT group which are consistent with the Dallas Heart Study [9] and the work from Daiana et al. [21], in which plasma PCSK9 levels were higher in diabetic subjects than in non-diabetic subjects.The mechanism underlying this association is not clear, but it is known that the PCSK9 gene was regulated by sterol regulatory element-binding protein 1 (SREBP1c) and hepatocyte nuclear factor 1-alpha (HNF1a) transcription factors, both of which are involved in insulin metabolism [21–23]. Costet et al. [22] reported that insulin can raise the expression of PCSK9 mRNA through regulating SREBP-1c. Miao et al. [24] found that hepatic PCSK9 mRNA and plasma PCSK9 protein levels were reduced by 55% to 75% in mice with liver-specific insulin receptor deficiency; 75% to 88% in mice with streptozotocin-induced diabetes; and 65% in ob/ob mice treated with antisense oligonucleotides against the insulin receptor. In our study, IGR and T2DM both had higher FINS concentrations than NGT subjects, and T2DM subjects had higher FINS concentrations than IGR (Table 1). We also found PCSK9 levels positively correlated with FINS (Table 2). Therefore, we speculated that the role of PCSK9 in the progression of type 2 diabetes and metabolic disorders could be mediated by insulin. However, the exact mechanism needs our further study.

It is now well-recognized that PCSK9 plays a key role in atherogenesis. Its effects on the development of atherosclerosis are mainly mediated via degradation of hepatic LDLR, which impairs the catabolism of LDL and subsequently causes hypercholesterolemia [25, 26]. Other data also suggest a regulatory role for PCSK9 on the catabolism of triglyceride-rich lipoproteins [25, 27]. Consistent with this, PCSK9 levels positively correlated with LDL-C, TG, and TC in all participants in our study (Table 2). Some clinical studies have observed a positive correlation between plasma PCSK9 levels and HDL-C [9, 21, 28], but in our study, we did not observe this correlation. Differences of race and study population selection may explain this discrepancy. Meanwhile, we also found that PCSK9 levels positively correlated with other CVD risk factors such as BMI and WC.

This present study had some limitations. First, the cross-sectional design is limited to clarify the causality between PCSK9 and 2-hPG. Second, serum PCSK9 levels in our study are lower than that from other studies, this may be explained by racial and ethnic differences. In addition, dietary differences could also account for the relatively lower PCSK9 levels in the Han Chinese as well. Third, we did not directly combine the PCSK9 with other inflammatory markers, such as the CRP, WBCC and PLT.

## Conclusions

Thus, we conclude that 1) elevated serum PCSK9 concentration could be a early biomarker for T2DM independent of other metabolic parameters; and 2) serum PCSK9 levels and 2-hPG are positively correlated in patients with metabolic diseases.

## Abbreviations

2-hPG: 2-h postchallenge plasma glucose
BMI: Body mass index
CVD: Cardiovascular disease
DBP: Diastolic blood pressure
FBG: Fasting blood glucose
HC: Hip circumference
HDL-C: High-density lipoprotein cholesterol
HOMA-IR: Homeostasis model assessment-estimated insulin resistance index
HR: Heart rate
IFG: Impaired fasting glucose
IGR: Impaired glucose regulation
IGT: Impaired glucose tolerance
LDL-C: Low-density lipoprotein cholesterol
LDLR: LDL receptor
NGT: Normal glucose tolerance
PCSK9: Proprotein convertase subtilisin/kexin type 9
SBP: Systolic blood pressure
T2DM: Type 2 diabetes
TC: Total cholesterol
TG: Triglyceride
WC: Waist circumference
WHR: Waist-to-hip ratio
